# Obituary

**Published:** 1903-05-15

**Authors:** 


### OBITUARY.

### DR. OTTO ARNOLD.

Dr. Arnold died at his home in Columbus, Ohio, April 1st.

Dr. Otto Arnold was born in Buffalo, N. Y., April 21st, 1854. His boyhood days were spent in Ironton, Ohio. He early evinced a desire for dentistry and was associated several years with Dr. Sloan in his home town. He graduated from the Ohio College of Dental Surgery in 1879. Returning to Ironton he opened up for himself, continuing there until 1886. In 1882 he was married to Jessie Johnson, who with one child, Louise, survives him. In March, 1886, he moved to Columbus and was in continuous practice until his death.

He was not only a successful practitioner but always took an active interest in his profession and had a wide acquaintance professionally, because of his literary and personal contributions to the professions.

For a few years after his graduation he was Demonstrator of Anatomy at his Alma Mater. In 1890 Dr. Arnold went as delegate from the National Association to the International Dental Congress, held at Berlin, Germany. In 1897 he was made dean of the Dental Department of the Ohio Medical University, resigning in 1901 because of his failing health. In 1902 the Ohio State Dental Society honored him with the presidency of that organization. As member of this society also the Columbus Dental Society, Mississippi Valley, Tri-State and National Societies, Dr. Arnold was active, willing and ready.

The past three or four winters were spent in the South and West seeking for health, and he had returned home from Arizona but two weeks before his death. On the first of April, 1903, he gave up the fight he had so valiantly waged.

His untimely death will be felt by a large circle of friends and acquaintances. His wife and daughter have the hearty sympathy of all his professional colleagues.

### DR. J. B. KIRK.

Dr. J. B. Kirk, of Columbus, Ohio, died February 25th at the age of 65 years. He was one of the oldest and best known practitioners of Columbus. While he was not much known outside of his own city and State, he was highly esteemed in his own city for his sterling character and professional ability. He was a member of the local and State societies and did what he could to uphold the reputation of his calling. He was associated in business with his son, Dr. H. M. Kirk, a member of the faculty of the Dental Department of the Ohio Medical University, to whom we extend the cordial sympathy of his professional confreres.

### DR. S. B. PALMER.

Dr. S. B. Palmer died March 30th at his home in Syracuse, N. Y., after an illness of three weeks. He had given up practice only a few weeks previous to the illness which caused his death. Dr. Palmer was one of the strongest advocates of what was called the "New Departure" in filling teeth. He attracted much attention a few years ago by his vigorous writings on this subject. He was a man who did his own thinking and never hesitated in promulgating his views, even though they were not in harmony with accepted ideas or scientific standards. He was highly esteemed by those who knew him best and had a host of warm personal and professional friends in his own State and the country at large. While it may be difficult to estimate the value of such a life to the profession, it is evident that his work has certainly helped to establish, even though negatively, better methods of practice.

